# Acknowledgement to Reviewers of *Brain Sciences* in 2015

**DOI:** 10.3390/brainsci6010003

**Published:** 2016-01-22

**Authors:** Brain Sciences Editorial Office

**Affiliations:** MDPI AG, Klybeckstrasse 64, CH-4057 Basel, Switzerland; brainsci@mdpi.com

The editors of *Brain Sciences* would like to express their sincere gratitude to the following reviewers for assessing manuscripts in 2015.

We greatly appreciate the contribution of expert reviewers, which is crucial to the journal’s editorial decision-making process. Several steps have been taken in 2015 to thank and acknowledge reviewers. Good, timely reviews are rewarded with a discount off their next MDPI publication. By creating an account on the submission system, reviewers can access details of their past reviews, see the comments of other reviewers, and download a letter of acknowledgement for their records. In addition, MDPI has launched a collaboration with Publons, a website that seeks to publicly acknowledge reviewers on a per journal basis. This is all done, of course, within the constraints of reviewer confidentiality. Feedback from reviewers shows that most see their task as a voluntary and mostly unseen work in service to the scientific community. We are grateful to our reviewers for the contribution they make.

Addis, Donna Rose
Angelini, Corrado
Baudry, Michel
Bear, Mark F.
Blum, Robert
Bozzi, Yuri
Brini, Marisa
Brown-Schmidt, Sarah
Ceroni, Fabiola
Conrad, Cheryl
Croxson, Paula L.
Dalrymple-Alford, John
Datta, Subimal
Daubney, Michael
Davalli, Angelo
Dos Santos, Ana Paula Marreilha
Duff, Melissa C.
Eisenbarth, Hedwig
Elmose, Mette
Feng, Yangzheng
Ferdinand, Nicola K.
Ferini-Strambi, Luigi
Fiore, Marco
Foland-Ross, Lara C.
Frye, Richard E.
Furtner, Julia
Gimpl, Gerald
Glavaski-Joksimovic, Aleksandra
Glazewski, Stanislaw
Göb, Eva
Greenberg, Tsafrir
Groenendaal, Floris
Gu, Zezong
Guerini, Franca R.
Gupta, Madhulika A.
Hach, Sylvia
Hagerman, Randi J.
Hasan, Bashar A. Shiekh
Hellewell, Sarah
Hoedlmoser, Kerstin
Hornickel, Jane
Howland, John G.
Hu, Xiaoling
Hu, Xiaosu
Hunt, Pamela
Hwang, Hyisung
Hwang, Tsong-Long
Im, Chang-Hwan
Innes-Brown, Hamish
Jensen, Randy L.
Johnson, Ann C.
Jones, Nicole M.
Kane, Cynthia J. M.
Kehoe, Elizabeth G.
Kennedy, Gerard
Kippin, Tod E.
Kohler, Mark
Koravand, Amineh
Kumar, R.
Kwack, KyuBum
Large, Edward
Leaver, Helen Anne
LeBourgeois, Monique
Lee, Seung-Koo
Leeming, R. J.
Lemola, Sakari
Lin, Yun-lian
Lopez, Marcelo F.
Lucock, Mark
MacNamara, Annmarie
Malmierca, Manuel S.
Manthey, John A.
Massoni, Sébastien
Maxwell, Judith
McGrath, Larry
Mellinger, Jurgen
Miskovic, Vladimir
Miyata, Seiji
Montag, Christian
Moser, Jason
Naseer, Noman
Neumann, Inga D.
Noll-Hussong, M.
Oechslin, Mathias S.
Omurtag, Ahmet
Paglialonga, Alessia
Pascual, Olivier
Payne, Jessica D.
Pino-Figueroa, Alejandro
Pinto-Duarte, Antonio
Plotz, Karsten
Pocivavsek, Ana
Prasad, Amit
Puschmann, Sebastian
Raes, Filip
Saletin, Jared
Saxena, Tarun
Schenck, Carlos
Seifert, Hilary
Semkovska, Maria
Shaffery, James
Sharma, Aruna
Sharp, Carla
Sheldon, Signy
Shen, Linlin
Shetake, Jai
Su, Dong-Ming
van Bel, Frank
Vervliet, Bram
Vidal, Cecilio J.
Vieira, Joana Barbosa
Viviani, Roberto
Voegeli, Géraldine
Witting, Paul
Wong, Jen D.
Yamamoto, Akihito
Yucel, Meryem

